# BMPR1a Is Required for the Optimal TGFβ1-Dependent CD207^+^ Langerhans Cell Differentiation and Limits Skin Inflammation through CD11c^+^ Cells

**DOI:** 10.1016/j.jid.2022.02.014

**Published:** 2022-03-14

**Authors:** Mathias Hochgerner, Thomas Bauer, Victoria Zyulina, Elisabeth Glitzner, Sarah Warsi, Joanne E. Konkel, Carmen Tam-Amersdorfer, Wanjun Chen, Stefan Karlsson, Maria Sibilia, Herbert Strobl

**Affiliations:** 1 Division of Immunology and Pathophysiology, Otto Loewi Research Center, Medical University of Graz, Graz, Austria; 2 Center for Cancer Research, Medical University of Vienna, Vienna, Austria; 3 Comprehensive Cancer Center, Vienna, Austria; 4 Division of Molecular Medicine and Gene Therapy, Lund Stem Cell Center, Lund University Hospital, Lund, Sweden; 5 Mucosal Immunology Section, National Institute of Dental and Craniofacial Research, National Institutes of Health, Bethesda, Maryland, USA; 6 The Lydia Becker Institute of Immunology and Inflammation, School of Biological Sciences, Faculty of Biology, Medicine and Health, The University of Manchester, Manchester, United Kingdom; 7 These authors contributed equally to this work

## Abstract

The cytokine TGFβ1 induces epidermal Langerhans cell (LC) differentiation from human precursors, an effect mediated through BMPR1a/ALK3 signaling, as revealed from ectopic expression and receptor inhibition studies. Whether TGFβ1–BMPR1a signaling is required for LC differentiation in vivo remained incompletely understood. We found that TGFβ1-deficient mice show defective perinatal expansion and differentiation of LCs. LCs can be identified within the normal healthy human epidermis by anti-BMPR1a immunohistology staining. Deletion of *BMPR1a* in all (vav^+^) hematopoietic cells revealed that BMPR1a is required for the efficient TGFβ1-dependent generation of CD207^+^ LC-like cells from CD11c^+^ intermediates in vitro. Similarly, BMPR1a was required for the optimal induction of CD207 by preformed major histocompatibility complex II–positive epidermal resident LC precursors in the steady state. BMPR1a expression is strongly upregulated in epidermal cells in psoriatic lesions, and BMPR1a^ΔCD11c^ mice showed a defect in the resolution phase of allergic and psoriatic skin inflammation. Moreover, whereas LCs from these mice expressed CD207, BMPR1a counteracted LC activation and migration from skin explant cultures. Therefore, TGFβ1–BMPR1a signaling seems to be required for the efficient induction of CD207 during LC differentiation in the steady state, and bone marrow–derived lesional CD11c^+^ cells may limit established skin inflammation through enhanced BMPR1a signaling.

## INTRODUCTION

Epidermal Langerhans cells (LCs) represent highly abundant evolutionary conserved dendritic cells (DCs) specifically located in stratified epithelia. The epidermal/epithelial microenvironment is known to play a key instructive role in LC lineage identity, as evidenced from transcriptomics (Capucha et al., 2015) and cell differentiation studies of human CD34^+^ hematopoietic progenitor cells (Régnier et al., 1998). Within epithelia, TGFβ family members TGFβ1 and BMP7 promote LC differentiation and regulate LC function. These proteins exhibit tightly controlled expression patterns. Specifically, TGFβ1 and BMP7 show opposite expression within epidermal keratinocyte layers, with BMP7 confined to basal layers and TGFβ1 expressed in suprabasal/outer layers (Yasmin et al., 2013). Moreover, BMP7 precedes TGFβ1 during prenatal epidermal development, suggesting that earliest LC precursors are exposed to BMP7 but not to TGFβ1 (Yasmin et al., 2013). Epidermal TGFβ family signaling is dysregulated during inflammation. Within the enlarged psoriatic epidermis, BMP7 is aberrantly expressed at high levels throughout all epidermal layers (Borek et al., 2020), whereas components of classical TGFβ signaling are reportedly repressed (Bobr al., 2010; Doi et al., 2003; Jiang et al., 2017; Wataya-Kaneda et al., 1996). In line with this, the lesional psoriatic epidermis (including LCs) exhibits strong constitutive-active BMP–SMAD1/5/8 activation, and BMP7 promotes the generation of LCs in vitro from CD34^+^ hematopoietic progenitor cells, exhibiting phenotypic characteristics of LCs found in psoriatic lesions (CD1c^+^CD206^+^CD1a^+^CD207^+^) (Borek et al., 2020).

Murine gene knockout (KO) studies revealed a critical role for classical/canonical TGFβ1–TGFβR1/ALK5 signaling in LC function. The group of M. Udey first showed that TGFβ1 is required for the presence of LCs in the murine epidermis (Borkowski et al., 1997, 1996). Subsequently, it was shown that mice lacking TGFβR1 in CD11c cells exhibit a marked loss of LCs after postnatal day (P) 3 (Kel et al., 2010) and that CD207-specific *Tgfbr2*-KO mice lack LCs in adult mice (Kaplan et al., 2007). Moreover, inducible deletion of either *TGFβR1* or *TGFβ1* in CD207^+^ cells resulted in a loss of LCs owing to enhanced LC activation and concomitant migration to draining lymph nodes (Bobr et al., 2012). Thus, classical (TGFβR1/ALK5–mediated) TGFβ1 signaling secures a nonactivated LC phenotype, essential for epidermal residency of LCs. Consistently, the absence of integrin-mediated TGFβ1 processing in mice lacking a_v_b_6_ or a_v_b_8_ in keratinocytes resulted in a loss of LCs in vivo (Mohammed et al., 2016). Together, these studies showed a key role of constitutive-active canonical TGFβ1 signaling in the maintenance of epidermal resident nonactivated LCs in the steady state.

In vitro, TGFβ1 induces LC differentiation from human hematopoietic progenitor cells (Strobl et al., 1996), CD14^+^ monocytes (Geissmann et al., 1998), and CD1c^+^ DCs (Ito et al., 1999; Martínez-Cingolani et al., 2014; Milne et al., 2015) when added to cytokine-supplemented culture media. Interestingly, evidence from in vitro studies suggested that TGFβ1-dependent LC lineage instruction relies on BMP receptor rather than on canonical TGFβΡ1/ALK5 signaling (Yasmin et al., 2013). BMPs are known to signal through heterodimeric BMP receptors (four type-1 and seven type-2 receptors) to activate SMAD1/5/8 (reviewed in Nickel and Mueller [2019]). Ectopic expression of BMPR1a/ALK3 but not ALK5 in CD34^+^ cells strongly augmented TGFβ1-dependent LC differentiation (Yasmin et al., 2013), and a soluble BMPR1a construct interfered with TGFβ1-mediated LC differentiation from CD1c^+^ blood DCs in vitro (Milne et al., 2015).

Whether TGFβ1–BMPR1a signaling is required for LC differentiation in vivo remained incompletely understood. Moreover, the mechanism underlying LC’s capacity to terminate/limit psoriatic skin inflammation remained poorly understood. BMPR1a-deficient mice do not survive after birth (Mishina et al., 1995). In a first attempt to study its role in LC biology, mice lacking BMPR1a in CD11c^+^ or CSFR1^+^ cells were generated (Yu et al., 2021). Networks of major histocompatibility complex II (MHCII)^+^ epidermal cells developed independently of BMPR1a in both models during steady state and inflammation, and BMPR1a was not required for the onset of skin inflammation (Yu et al., 2021). In this study, we show that BMPR1a is required for the efficient TGFβ1-dependent LC differentiation in vitro and that both TGFβ1 and BMPR1a are required for the optimal intraepidermal differentiation of CD207^+^ LCs. Moreover, BMPR1a was required in CD11c^+^ cells for terminating established skin lesions.

## RESULTS

### TGFβ1 drives postnatal LC differentiation, and neonatal LC precursors are partially dependent on TGFβ1 in vivo

**Figure 1.**
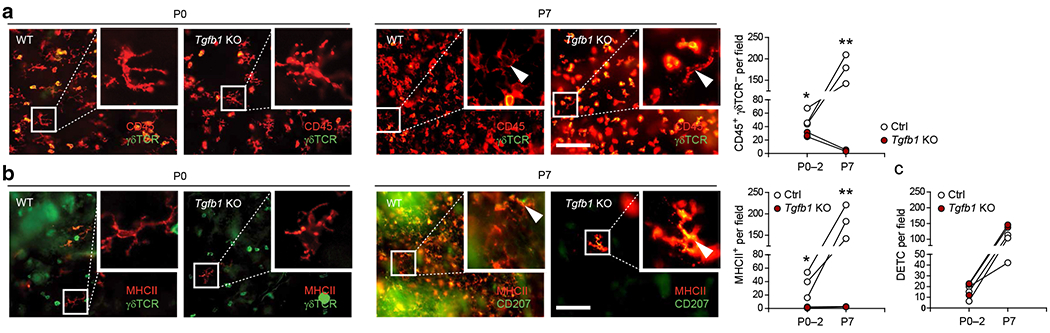
Epidermal LC precursors are only partially dependent on TGFβ1 prenatally, and TGFβ1 drives normal LC differentiation from these precursor cells in vivo. (**a**) Epidermal sheets WT and *Tgfβ1*-KO mice at P0 and P7 stained for γδTCR (green) and CD45 (red) to identify LC precursors (CD45^+^, γδTCR^−^) and its quantification. (**b**) Epidermal sheets from WT and *Tgfβ1*-KO mice at P0 and P7 stained for MHCII (red) and γδTCR or CD207 (green) to identify early LCs (MHCII^+^, γδTCR^−^) as indicated and its quantification. (**c**) Quantification of γδTCR^+^ CD45^high^ DETCs at the indicated time points. Each dot represents one mouse (n = 3 per group and time point). Arrowheads indicate an LC or its precursor. Inlets represent enlarged areas as indicated in the picture. P0–2 represents pooled data from mice at the day of birth (P0), P1, and P2. Graphs are shown as mean ± SEM. *P < 0.05 and **P < 0.01 are determined by Student’s *t*-test. Bar = 100 μm. Ctrl, control; DETC, dendritic epidermal T cell; KO, knockout; MHCII, major histocompatibility complex II; LC, Langerhans cell; P, postnatal day; WT, wild type.

Perinatal LC differentiation in TGFβ1-deficient mice has not been fully studied. We monitored LCs in epidermal sheets from *Tgfβ1*-KO mice at birth and during the first week of life (P0 and P7) (Figure 1). We observed that *Tgfβ1*-KO mice display a slightly reduced network of CD45^+^ γδTCR^−^ dendritic-shaped LC precursor cells in the epidermis at birth (Figure 1a). These cells lack MHCII expression, with the exception of rare scattered cells (Figure 1b). Monitoring postpartum LC differentiation revealed a key reliance of P7 LCs on TGFβ1 (Figure 1b, right panel). Specifically, although wild-type (WT) mice exhibit strong increases in the frequencies of both epidermal CD45^+^ γδTCR^−^ and MHCII^+^ cells from P0 to P7, these cells could hardly be detected in *Tgfb1*-KO mice at P7 (Figure 1a and b). For comparison, no detectable changes were observed in the dendritic epidermal T-cell network at the indicated time points (Figure 1c). These data suggest that TGFβ1 is the dominant driver of postpartum LC differentiation. Moreover, TGFβ1 is partially required for the neonatal presence of dendritic-shaped epidermal LC precursors and for MHCII expression by these cells.

### TGFβ1-dependent in vitro LC differentiation requires BMPR1a for optimal differentiation of LC-like cells

**Figure 2.**
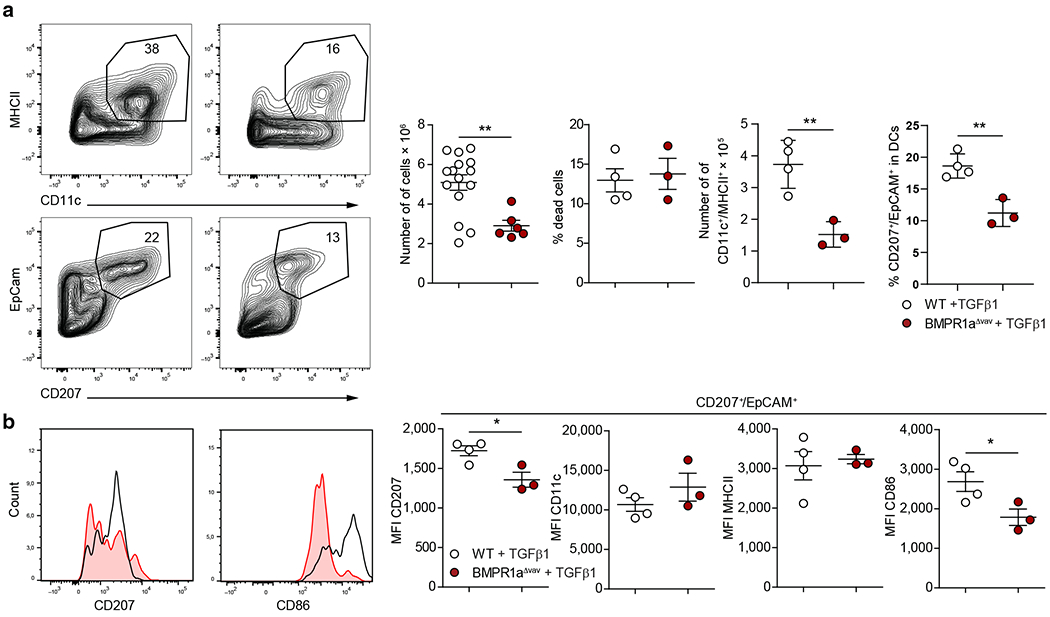
TGFβ1-dependent in vitro LC generation requires BMPR1a for optimal differentiation of LC-like cells. (**a**) FACS plots represent bone marrow of WT and BMPR1a^Δvav^ mice after culture for 5 d in the presence of GM-CSF and TGFβ1 as indicated. Cells were enumerated and analyzed for the indicated markers. Gated MHCII^+^CD11c^+^ cells from the cultures described earlier are analyzed for CD207 versus for EpCam. (**b**) Gated CD207^+^EpCam^+^ are analyzed for the indicated marker molecules; MFI values are shown. A representative histogram for CD207 is shown (black line = WT, red line = BMPR1a^Δvav^ ). Each dot represents bone marrow cells from one mouse. n ≥ 3. Values are shown as mean ± SEM. *P < 0.05 and **P < 0.01 are determined by Student’s *t*-test. d, day; DC, dendritic cell; LC, Langerhans cell; MFI, mean fluorescence intensity; MHCII, major histocompatibility complex II; WT, wild type.

Given the above observations mentioned earlier that TGFβ1 is the dominant driver of postpartum LC differentiation, we next asked whether BMPR1a is required for TGFβ1-mediated LC differentiation. BMPR1a is one of the four type-1 BMP receptors and is known to heterodimerize with the type-2 receptor BMPR2 to transmit signals through BMPs and other TGFβ family ligands. In addition to BMPR2, two other type-2 BMP receptors are known (Nickel and Mueller, 2019). We generated mice lacking BMPR1a throughout the hematopoietic system using the vav-cre promotor (BMPR1a^Δvav^). Adding TGFβ1 to GM-CSF–supplemented murine bone marrow (BM) cultures of WT mice redirects CD11c^+^ DC differentiation into CD207^+^ EpCAM^+^ LC-like DCs (i.e., BM-derived LCs). Cultures initiated with BM from BMPR1a^Δvav^ mice exhibited strongly diminished total cell yields and the numbers of CD11c^+^MHCII^+^ cells (Figure 2a and Supplementary Figure S1a). This effect was not accompanied by enhanced cell death (Figure 2a). The BM-derived LC generation cultures initiated from BMPR1a^Δvav^ mice also displayed substantially reduced percentages of CD207^+^EpCAM^+^ cells among gated CD11c^+^MHCII^+^ cells compared with that in parallel cultures initiated from WT control mice (Figure 2a). Moreover, gated CD207^+^EpCAM^+^ cells exhibited diminished CD207 and CD86 expression mean expression densities (Figure 2b). Therefore, BMPR1a is functionally required for the optimal generation of TGFβ1-induced LC-like cells in vitro.

### BMPR1a is required for the efficient induction of CD207 during postnatal LC differentiation in vivo

**Figure 3.**
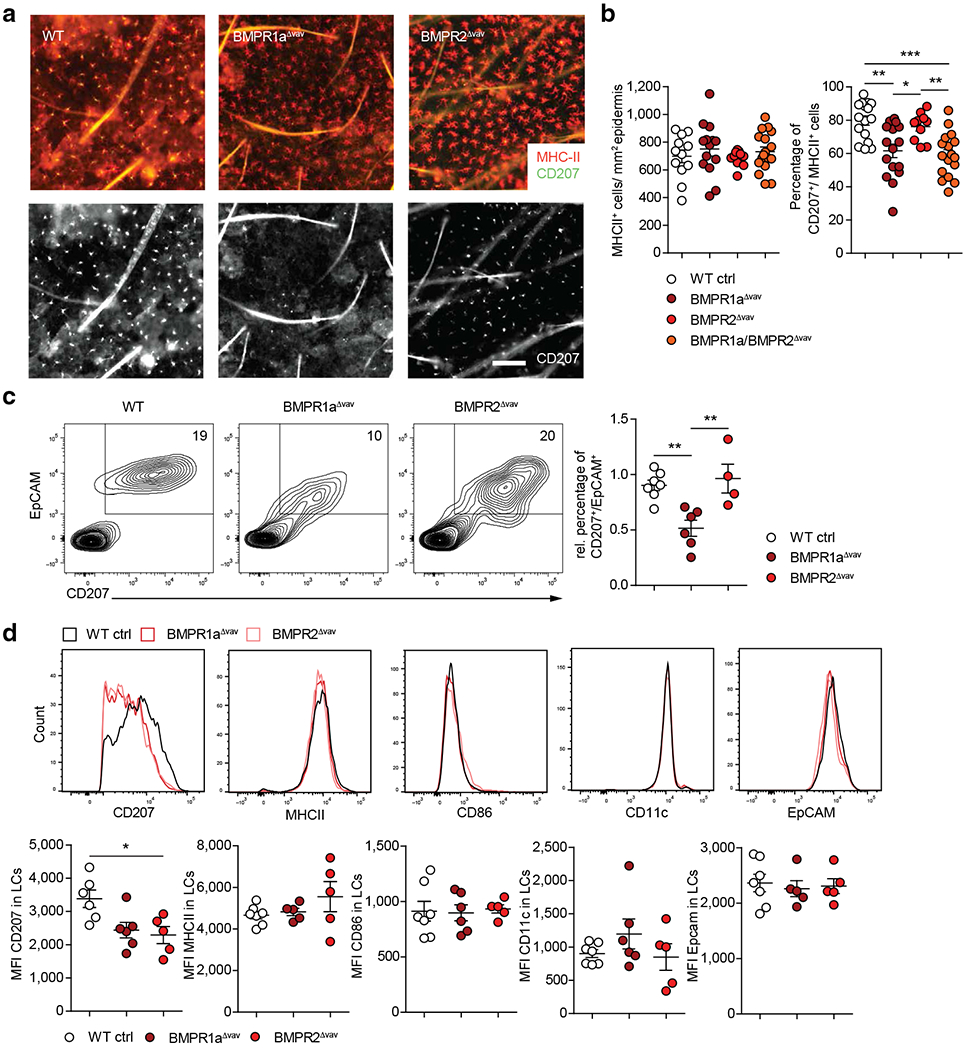
BMPR1a promotes the induction of CD207 during perinatal LC differentiation in vivo. (**a**) Epidermal sheets from WT, BMPR1a^Δvav^, and BMPR2^Δvav^ mice were stained for MHCII (red, upper panel) and CD207 (white, lower panel) to identify LCs. (**b**) MHCII^+^ and CD207^+^ cells from epidermal sheets were enumerated. The percentage of CD207^+^ cells among MHCII^+^ cells is shown in the right graph. (**c**) Representative FACS plot of epidermal cell suspensions from WT, BMPR1a^Δvav^, and BMPR2^Δvav^ mice and its relative enumeration. (**d**) CD207^+^ EpCam^+^ cells from the cultures mentioned earlier were gated, the MFI of indicated markers was analyzed, and representative histograms are shown. Each dot represents one mouse. n ≥ 4. Values are shown as mean ± SEM. *P < 0.05, **P < 0.01, and ***P < 0.001 as determined by Student’s *t*-test. Bar = 100 μm. Ctrl, control; LC, Langerhans cell; MFI, mean fluorescence intensity; MHCII, major histocompatibility complex II; rel., relative; WT, wild type.

We next studied LCs from mice lacking BMPR1a (BMPR1a^Δvav^) or BMPR2 (BMPR2^Δvav^) in hematopoietic cells in vivo. Deletion of *BMBPR1a* or *BMPR2* did not affect the numbers of epidermal MHCII^+^ dendritic-shaped cells. However, MHCII^+^ cells from BMPR1a-deficient mice lacked or showed markedly reduced CD207 expression (Figures 3a and b and Supplementary Figure S1b). Moreover, although the frequencies of CD207^+^ cells remained unchanged in WT compared with that in BMPR2^Δvav^ mice, the deletion of *Bmpr2* in BMPR1a-deficient mice (double-KO mice) led to a drop in LC numbers (i.e., similar to that in BMPR1a single-deficient mice) (Figure 3b). FACS analysis of epidermal single-cell suspensions confirmed the observed reduction in the percentage of CD207^+^EpCAM^+^ LCs in epidermal cell suspensions prepared from BMPR1a^Δvav^ mice (Figure 3c). Gated CD207^+^EpCAM^+^ cells from both BMPR1a^Δvav^ and BMPR2^Δvav^ mice showed reduced expression densities for CD207. Expression densities for MHCII, CD11c, EpCAM, and CD86 by gated CD207^+^EpCAM^+^ cells did not differ significantly between WT and gene KO mice (Figure 3d). In conclusion, hematopoietic cells lacking the type-1 BMP receptor BMPR1a exhibit normal numbers of MHCII^+^ epidermal cells, but these cells lacked or showed reduced CD207 expression. Moreover, FACS analyses revealed that CD207^+^ LCs from BMPR2^Δvav^ mice showed reduced CD207 expression densities.

### Human BMPR1a expression by epidermal cells in steady versus in psoriatic lesions

**Figure 4.**
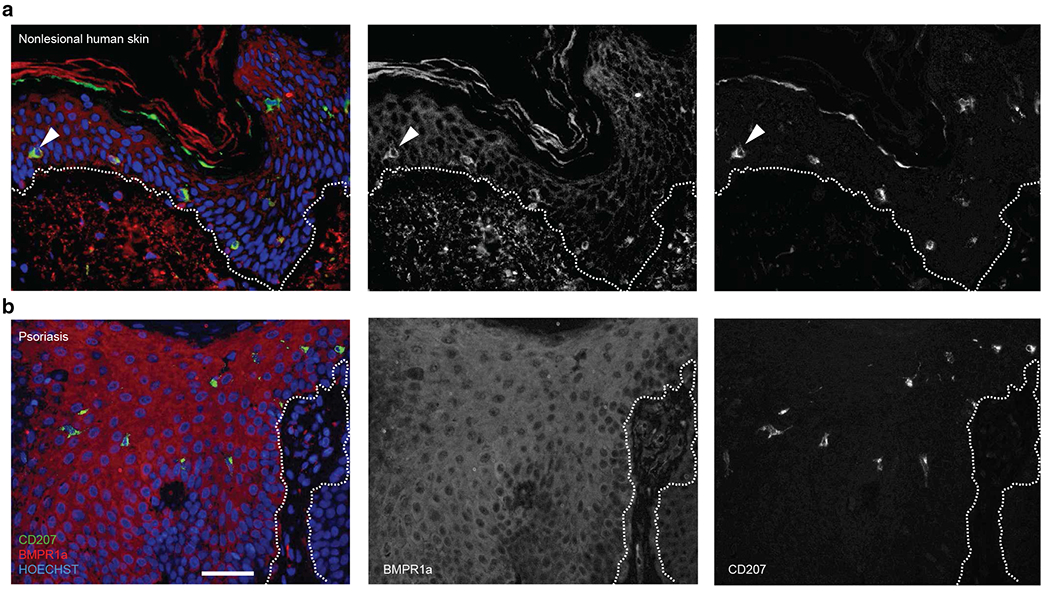
Human LCs express BMPR1a and enhanced BMPR1a expression by lesional psoriatic epidermal cells. Representative immunohistology for BMPR1a and CD207 expression from (**a**) nonlesional and (**b**) lesional skin from a patient with psoriasis. The white dotted line represents the epidermal/dermal junction. Arrowhead indicates an LC. Data are representative of five independent patient samples. LC, Langerhans cell.

We subsequently studied BMPR1a expression by epidermal cells in steady state versus in inflammation using immunohistology of human skin. BMPR1a is expressed by CD207^+^ LCs at higher levels than by adjacent keratinocytes, allowing to identify LCs on the basis of BMPR1a expression (Figure 4a), similarly as previously observed for BMPR2 (Borek et al., 2020). Epidermal cells from the enlarged lesional psoriatic epidermis showed stronger BMPR1a expression than those from nonlesional human skin (Figure 4b). In harmony with these findings, anti-SMAD1/5/8 immunohistology previously revealed strong reactivity with lesional epidermal cells and LCs, in keeping with the aberrant high epidermal BMP7 expression in psoriatic lesions (Borek et al., 2020).

### Functional in vivo implications of BMPR1a signaling in LCs

**Figure 5.**
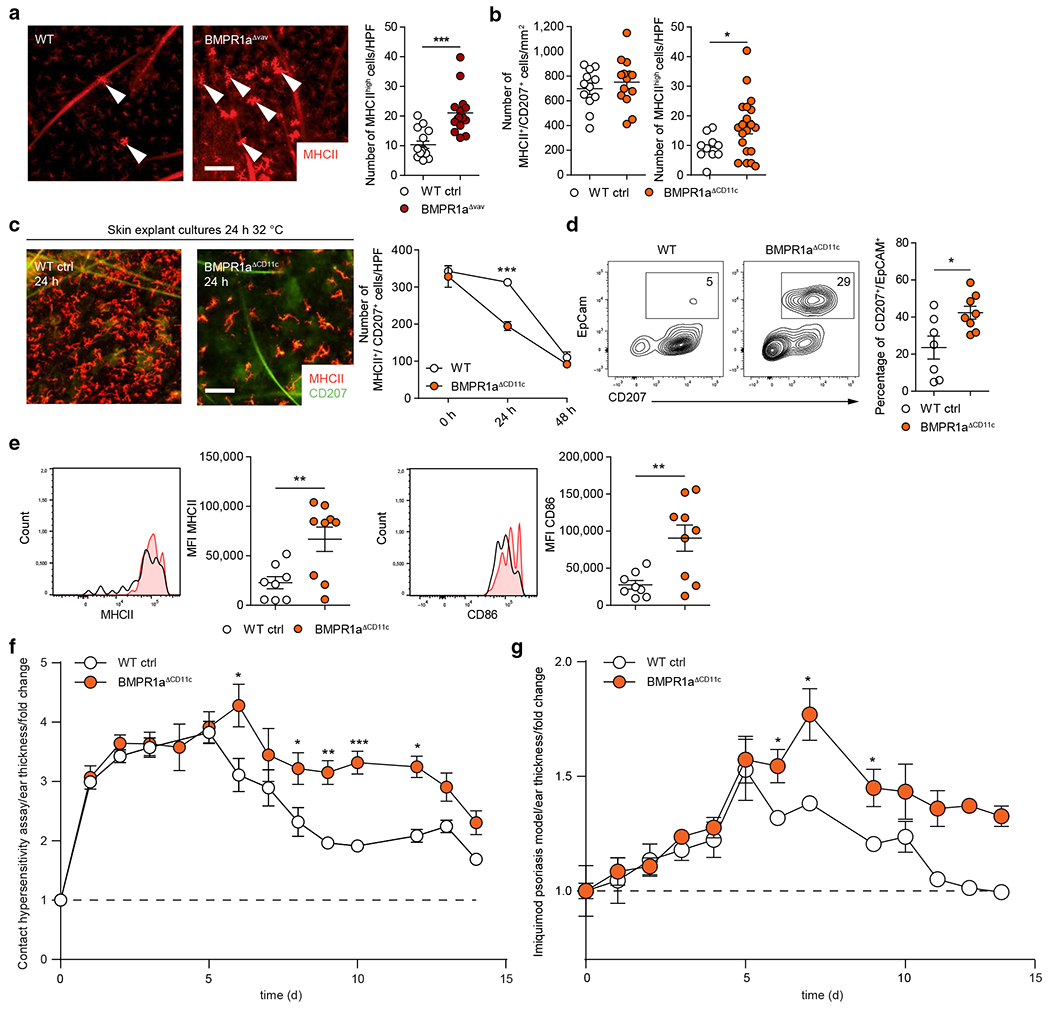
Deletion of BMPR1a in CD11c^+^ cells affects LC function in vivo but does not affect perinatal LC differentiation. (**a**) Epidermal sheets from WTand BMPR1a^Δvav^ mice stained for MHCII (red). MHCII^high^ cells as indicated by the arrowhead were enumerated and depicted in the right graph. (**b**) Epidermal sheets from WT and BMPR1a^ΔCD11c^ mice stained for MHCII (red) and CD207 (green) to identify LCs. MHCII^+^ and CD207^+^ cells from epidermal sheets were enumerated (left graph). The number of MHCII^high^ cells from the same epidermal sheets is shown in the right graph. (**c**) Ear halves (i.e., skin explants) from WT and BMPR1a^AΔCD11c^ mice were floated on a complete medium for 24 and 48 h. Epidermal sheets were analyzed for LC distribution (MHCII^+^ and CD207^+^ cells) and enumerated. Representative pictures from 24 h are shown on the left, graph from 24 h and 48 h is shown on the right. (**d**) Representative FACS plots and enumeration of emigrated cells from the skin explant cultures. (**e**) Analysis of emigrated gated CD207^+^EpCAM^+^ cells (gated as shown in **d**) for MHCII and CD86 expression. Representative histograms are shown **d**. Each dot represents data from one mouse. (**f**) Contact hypersensitivity assay was performed on WT and BMPR1a^ΔCD11c^ mice using DNFB, and ear swelling was monitored for the indicated time points. (**g**) Imiquimod skin inflammation assay was performed on WT and BMPR1a^ΔCD11c^ mice. Ear swelling was monitored for the indicated time points; shown as mean ± SEM. n ≥ 4 per group and time point. The graph represents data from at least two independent experiments. *P < 0.05, **P < 0.01, and ***P < 0.001 are determined by Student’s *t*-test for each time point. Bar = 100 μm. ctrl, control; d, day; h, hour; HPF, high power field; LC, Langerhans cell; MFI, mean fluorescence intensity; MHCII, major histocompatibility complex II; WT, wild type.

In subsequent experiments, we asked whether BMPR1a is functionally involved in LC maturation/activation. First, we observed an LC network with scattered LCs displaying highly elevated levels of MHCII from BMPR1a^Δvav^ mice, indicating the immunological activation of these cells without apparent instability of the LC network (Figure 5a, arrowhead). These MHCII^high^ cells can also be observed in WT mice, but significantly more of these cells were found in the BMPR1a^Δvav^ mice (Figure 5a, right graph). In contrast to BMPR1a^Δvav^ mice, BMPR1a^ΔCD11c^ mice contained similar numbers of MHCII^+^CD207^+^ LCs as observed in WT mice (Figure 5b). Nevertheless, similarly as observed for BMPR1a^Δvav^ mice, they showed elevated numbers of scattered MHCII^high^ epidermal LCs relative to WT control mice (Figure 5b, right graph). Because BMPR1a^ΔCD11c^ mice contain undisturbed numbers of CD207^+^ LCs, we used these mice for functional analyses. We recently showed that BMPR1a^ΔCD11c^ mice show enhanced psoriasis-like skin inflammation in response to toll-like receptor 7 ligand imiquimod (IMQ) (Sconocchia et al., 2021). On the basis of these observations, we studied LCs in more detail. In vivo, LCs exhibit a constant turnover and get replenished by local LC proliferation and by immigration of BM-derived cells (reviewed in Hovav, [2018]). Skin explant cultures were previously shown to allow kinetic studies on LC emigration, mimicking stress conditions to the epidermal LC niche (Bauer et al., 2021). Although similar numbers of LCs from both WT and BMPR1a^ΔCD11c^ mice migrated within 48 hours, LCs from BMPR1a^ΔCD11c^ mice displayed a faster migration kinetic than LCs from WT controls (resulting in differences at 24 hours) (Figure 5c and d). LC egress from the epidermis is paralleled by the upregulation of activation markers such as MHCII and CD86. Nevertheless, migrated LCs from BMPR1a^ΔCD11c^ mice collected from the medium exhibited on average higher MHCII and CD86 expression densities than those from WT mice (Figure 5e and Supplementary Figure S1c). Therefore, BMPR1a counteracts LC activation and slows migration during conditions of epidermal stress.

### Late-acting anti-inflammatory effect of BMPR1a in CD11c^+^ cells in vivo

In subsequent experiments, we tested the consequences of epidermal stress in vivo. After skin sensitization with DNFB, BMPR1a^ΔCD11c^ mice were challenged, and ear swelling was subsequently monitored during the inflammatory response (Figure 5f). After an unaltered contact hypersensitivity reaction 24 hours after the challenge, skin inflammation persisted and did not resolve rapidly in BMPR1a^ΔCD11c^ mice. IMQ-induced psoriatic skin inflammation displayed similar kinetics in BMPR1a^ΔCD11c^ mice and WT mice until day 5, as also observed using independently generated BMPR1a^ΔCD11c^ mice (Yu et al., 2021). However, starting on day 6 after IMQ treatment, BMPR1a^ΔCD11c^ mice exhibited an enhanced inflammatory reaction (Figure 5g). In line with these observations, BMPR1a^Δvav^ mice also exhibited increased inflammation beyond day 5 in the IMQ model (Supplementary Figure S2a). Conversely, BMPR2^Δvav^ mice did not differ from WT controls (Supplementary Figure S2b).

## DISCUSSION

The TGFβ family ligands TGFβ1 and BMP7 show tightly regulated inverse expression patterns within the epidermal microenvironment during steady state and inflammation and are known to promote LC differentiation and to regulate LC function. Two major signaling cascades are known, that is, canonical TGFβ and BMP signaling, induced by TGFβ1 and TGFβ1/BMPs, respectively. Whether BMP receptor signaling is of relevance for LC differentiation in vivo and whether it is functionally involved in LC’s immunoregulatory role remained poorly understood. In this study, we showed that TGFβ1 signals through BMPR1a to induce efficient CD207^+^ LC differentiation from their precursors. In addition, we propose a model whereby enforced BMP signaling in DCs/LCs is critical for terminating skin inflammation.

We found that TGFβ1–BMPR1a signaling promotes CD207^+^ LC differentiation from pregenerated DCs. This notion is supported by in vitro and in vivo data. We added TGFβ1 to GM-CSF–supplemented BM-DC cultures and studied the acquisition of LC characteristics. In these cultures, BMPR1a was required for the optimal TGFβ1-dependent generation of LC-like cells. In vivo analysis subsequently confirmed that BMPR1a is specifically required for the efficient intraepidermal acquisition of CD207 expression by preformed MHCII^+^ epidermal resident LC precursors. Together with the data from TGFβ1-deficient mice presented in this study, these findings indicate that TGFβ1–BMPR1a signaling promotes the differentiation of CD207^+^ LCs from MHCII^+^ epidermal precursors in the steady state. Our data are in line with previous in vivo findings showing that low levels of BMPR1a expression by MHCII^+^ epidermal LC precursors (owing to Cbfβ2 deficiency) correlate with defective terminal LC differentiation (Tenno et al., 2017) and that dorsomorphin (an inhibitor of ALK2/3/6) interferes with mucosal LC regeneration by arresting LC precursors at an Epcam^+^CD207^−^ stage (Capucha et al., 2015).

We previously identified TGFβ1 to be critically required for CD207 expression induction and LC differentiation from human monocyte progenitor cells in vitro and showed that TGFβ1-dependent LC differentiation is strongly promoted by the ectopic expression of BMPR1a/ALK3 in human CD34^+^ progenitor cells (Strobl et al., 1996; Yasmin et al., 2013). In this study, we showed that murine BM-derived LC precursors require BMPR1a for efficient proliferation and differentiation in vitro in response to TGFβ1. In combination, these findings showed that BMPR1a signaling plays a conserved role in TGFβ1-dependent LC differentiation from their precursor cells in vitro.

Although epidermal seeding of CD45^+^ LC precursors occurred largely independent of TGFβ1, the induction of MHCII by these precursors and the expansion of day 7 LC numbers depended on TGFβ1. BMP7 precedes TGFβ1 during prenatal epidermal development, and BMP7-deficient mice show substantially reduced numbers of epidermal LC precursors at birth, with these cells lacking typical dendritic morphology (Yasmin et al., 2013). Our data indicate that BMP7 and TGFβ1 mediate prenatal and perinatal LC differentiation independently of BMPR2/BMPR1a receptor complexes, confirming and extending recent observations (Yu et al., 2021). Therefore, future studies are required to shed light on the molecular mechanism governing LC precursor cell proliferation and differentiation in response to TGFβ family ligands.

In contrast to BMPR1a^Δvav^ mice, BMPR1a^ΔCD11c^ mice contained undisturbed numbers of epidermal CD207^+^ LCs. These observations might be explained by the fact that the CD11c promoter drives target gene deletion only after the initial start of LC differentiation. However, early deletion of *Bmpr1a* in myeloid cells in BMPR1a^ΔCSFR1^ mice did not similarly result in diminished expression of CD207 (Yu et al., 2021). Developmental differences of LCs might underly these discrepancies. Whereas Yu et al. (2021) analyzed LCs repopulating the skin 2 weeks after UV treatment, in this study, we analyzed steady-state LCs. Corresponding data on CD207 expression by steady-state LCs were not presented by Yu et al. (2021). Interestingly, steady-state versus BM-derived LC differentiation pathways differ in their requirement for ID2, a factor inducing downstream of TGFβ1 during LC differentiation in vitro (reviewed in Hieronymus et al. [2015]). Therefore, a signaling node comprising TGFβ1–BMPR1a–ID2 might promote LC differentiation in the steady state but not during inflammatory LC replenishment from BM-derived cells. Futures studies are required to analyze this possibility. Another limitation of our study is that we did not resolve the question of whether LC precursors from TGFβ1 deficient mice truly fail to differentiate, die, or emigrate out of the epidermis.

In line with a role for BMPR1a signaling in LC differentiation, anti-BMPR1a (this study) and anti-BMPR2 (Borek et al., 2020) stainings revealed bright positivity of LCs in the steady state, even enabling the identification of LCs among epithelial cells in tissue sections. Nevertheless, despite this observation, our study confirms previous data showing that BMPR1a is not required for the maintenance of LC networks in vivo (Yu et al., 2021). In conclusion, canonical TGFβ1–TGFβR1/ALK5 signaling rather than BMPR1a signaling is the dominant pathway securing LC network maintenance in the steady state. Nevertheless, BMPR1a deficiency in hematopoietic cells and CD11c^+^ cells resulted in the enhanced presence of scattered MHCII^high^ epidermal cells, indicative of activated LCs. Moreover, LCs lacking BMPR1a migrated with faster kinetics in skin explant cultures, with migrated LCs showing enhanced expression of DC maturation markers.

Immunohistology revealed higher frequencies of scattered MHCII^high^ cells (total cellular MHCII expression) among epidermal MHCII^+^ cells in mice lacking BMPR1a than in WT mice. Conversely, FACS analysis revealed similar expression densities of MHCII by LCs from BMPR1a-deficient versus from WT mice. These discrepancies can potentially be explained by activation-induced MHCII upregulation by LCs during the preparation of epidermal single-cell suspensions.

An important observation of our study was that BMPR1a is strongly upregulated in epidermal cells in psoriatic lesions, corroborating enhanced BMP7 expression and activated BMP receptor signaling in lesional keratinocytes and LCs (Borek et al., 2020). Skin inflammation and psoriatic epidermal thickening are accompanied by the neorecruitment of inflammation-associated LCs from circulating myelomonocytic precursor cells. These cells differ from steady-state LCs phenotypically (i.e., induced expression of CD1c, CD206, and toll-like receptor 2), and they showed higher mitotic activity (Borek et al., 2020). In addition, in the murine model, BM-derived LCs are known to populate the inflamed psoriatic epidermis at later stages during IMQ-induced inflammation (Glitzner et al., 2014). We previously performed cell ablation studies showing that although LCs are dispensable for the onset of psoriatic cutaneous lesion formation, they are critically required during the resolution phase of psoriatic inflammation. Epidermal resident LCs in psoriatic lesions exhibited strong BMP signaling, as revealed from anti-phosphorylated SMAD1/5/8 stainings (Borek et al., 2020). These cells might correspond to the aforementioned ID2-independent BM-derived LCs. In line with a role for BMPR1a in LC’s capacity to terminate skin inflammation, the effect of BMPR1a deficiency in CD11c^+^ cells on inflammation was only detectable from day 6 onward after IMQ treatment. However, a limitation of our study is that we did not specifically delete *Bmpr1a* in LCs. Therefore, we cannot rule out that the observed changes are due to CD11c^+^ cells lacking LC characteristics, including conventional DCs or dermal macrophages. Future studies are needed to analyze BMPR1a expression by these cells and to specifically delete *Bmpr1a* in LCs. It will be particularly interesting to analyze whether strong BMPR1a signaling as observed in BMP7^high^ epidermal murine psoriatic lesions might render BM-derived LCs capable of resolving established psoriatic lesions.

The late anti-inflammatory effects of BMPR1a in CD11c^+^ cells or vav^+^ cells in skin inflammation models might be at least partially mediated by DC/LC-mediated induction of regulatory T cells. We previously observed decreased numbers of skin-resident regulatory T cells in BMPR1a^ΔCD11c^ mice. Moreover, BMP7-derived LCs (phenotypically resembling LCs from psoriatic lesions) exceeded TGFβ1-derived LCs (phenotypically similar to LCs in the steady state) in promoting regulatory T-cell generation from naive T cells (Sconocchia et al., 2021). In addition, BMP7 synthesized by BMP7-derived LCs might promote regulatory T-cell generation through BMPR1a signaling in T cells in vitro (Sconocchia et al., 2021). Future studies are needed to further analyze this possibility and to see whether a similar mechanism might be operative in other models of inflammation and cancer immune evasion.

Our data support the concept that CD207^+^ LC differentiation occurs locally within the epidermal microenvironment in response to BMPR1a signaling. Interestingly, LC differentiation shows molecular similarities with mesenchymal-to-epithelial transition signaling during embryogenesis (Capucha et al., 2015; Hieronymus et al., 2015), a finding that is in line with those of previous studies describing that BMP signaling induces mesenchymal-to-epithelial transition in other cell types (Samavarchi-Tehrani et al., 2010; Zeisberg et al., 2005). Our data indicate that LC mesenchymal-to-epithelial transition occurs in response to epithelial signals. Future studies should further dissect BMPR1a versus TGFβR signaling input in LC function. For example, we previously identified induction of the TYRO3-AXL-MERTK receptor AXL downstream of TGFβ1 signaling during LC differentiation from monocytes and CD34^+^ cells (Bauer et al., 2012), with AXL being inhibited by specific interference with classical TGFβR/ALK5 signaling. Interestingly, AXL deficiency resulted in a loss of regulatory DCs in cancer models (Maier et al., 2020).

In conclusion, within the steady-state epidermis, TGFβ1 can be detected in suprabasal and outer epidermal layers. According to the data presented in this study, TGFβ1–BMPR1a seems to promote CD207^+^ LC differentiation in the steady state. Conversely, during psoriatic skin inflammation, BMP7–BMPR1a signaling is upregulated. Our data support a hypothetical model, whereby strong BMP signaling licenses BM-derived inflammation-associated LCs/DCs to limit established skin inflammation.

## MATERIALS AND METHODS

### Mice

*Tgfβ1*-KO and *Bmpr1a*^fl/fl^ mice were previously described (Borkowski et al., 1996; Yuhki et al., 2004). *Bmpr2*^fl/fl^ mice were obtained from Mutant Mouse Resource and Research Center (St. Louis, MO). BMPR1a^Δvav^/BMPR1a^ΔCD11c^ and BMPR2^Δvav^ mice were generated by crossing with vav-cre or CD11c-cre lines. The aforementioned mice were bred and maintained in the facilities of the Medical University of Vienna (Vienna, Austria) in accordance with institutional policies and federal guidelines. All mice had access to food and water ad libitum. Animal experimental procedures were approved by the Animal Experimental Ethics Committee of the Medical University of Vienna and the Austrian Federal Ministry of Science and Research (animal license numbers GZ 66.009/124-BrGT/2003, GZ 66.009/109-BrGT/2003, GZ BMWF-66.009/0073-II/10b/2010, GZ BMWF-66.009/0074-II/10b/2010, GZ BMWFW-66.009/0200-WF/II/3b/2014, GZ BMWFW-66.009/0199-WF/II/3b/2014).

### Preparation of epidermal sheets, epidermal single-cell suspensions, and analysis

See the Supplementary Materials and Methods.

### BM-derived LC generation

BM cells were isolated from the tibias and femurs of mice aged 6–8 weeks. Cells were then differentiated for 7 days in RPMI containing 5% fetal bovine serum. Differentiation media was supplemented with 20 ng/ml murine GM-CSF (≥2 × 10^7^ U/mg; PeproTech, London, United Kingdom) for DC differentiation. Where indicated, 0.5 ng/ml human TGFβ1 (2.5 × 10^4^ U/mg; R&D Systems, Minneapolis, MN) was added to the cultures for LC generation.

### Skin explant cultures

See the Supplementary Materials and Methods.

### IMQ skin inflammation assay

Mice were treated daily with a 5% cream formulation of IMQ (Aldara, Meda AB, Stockholm County, Sweden) on the backside of the ear for 7 days and left untreated thereafter for 7 additional days to study the resolution of skin inflammation.

### Contact hypersensitivity assay

Mice were shaved, and their abdomens were exposed to 0.5% DNFB (Sigma-Aldrich, St. Louis, MO) in 4:1 acetone/olive oil (40 μl). After 5 days (sensitization phase), the baseline ear thickness was measured using a dial thickness gauge (Mitutoyo, Kawasaki, Japan), and the left ear was treated on both sides epicutaneously with a 0.3% DNFB solution in acetone/olive oil (20 μl; elicitation phase). Ear thickness was measured at the indicated time points for 14 days as an indicator of cutaneous inflammation.

### Purification and in vitro culture of human hematopoietic cells

See the Supplementary Materials and Methods.

### Immunofluorescent microscopy, cytokines, reagents, and flow cytometry

See the Supplementary Materials and Methods.

### Patient samples and statistics

Ethics approval (EK700/2009) was obtained from the Medical University of Vienna (Vienna, Austria) Institutional Review Board for these studies. Human subjects and donors of human blood have provided written informed consent. See the Supplementary Materials and Methods for details.

### Data availability statement

No dataset was created in this study. All data are available from the corresponding author.

## Supplementary Material

1

## Abbreviations:

BM: bone marrow
DC: dendritic cell
IMQ: imiquimod
KO: knockout
LC: Langerhans cell
MHCII: major histocompatibility complex II
P: postnatal day
WT: wild type

## References

[R1] Bauer T, Gubi D, Klufa J, Novoszel P, Holcmann M, Sibilia M. Ex-vivo skin explant culture is a model for TSLP-mediated skin barrier immunity. Life (Basel) 2021;11:1237. 34833113 10.3390/life11111237 PMC8623134

[R2] Bauer T, Zagórska A, Jurkin J, Yasmin N, Köffel R, Richter S, Identification of Axl as a downstream effector of TGF-β1 during Langerhans cell differentiation and epidermal homeostasis. J Exp Med 2012;209:2033–47. 23071254 10.1084/jem.20120493 PMC3478937

[R3] Bobr A, Igyarto BZ, Haley KM, Li MO, Flavell RA, Kaplan DH. Autocrine/paracrine TGF-β1 inhibits Langerhans cell migration. Proc Natl Acad Sci USA 2012;109:10492–7. 22689996 10.1073/pnas.1119178109 PMC3387113

[R4] Bobr A, Olvera-Gomez I, Igyarto BZ, Haley KM, Hogquist KA, Kaplan DH. Acute ablation of Langerhans cells enhances skin immune responses. J Immunol 2010;185:4724–8. 20855870 10.4049/jimmunol.1001802 PMC3050031

[R5] Borek I, Köffel R, Feichtinger J, Spies M, Glitzner-Zeis E, Hochgerner M, BMP7 aberrantly induced in the psoriatic epidermis instructs inflammation-associated Langerhans cells. J Allergy Clin Immunol 2020;145:1194–207.e11. 31870764 10.1016/j.jaci.2019.12.011

[R6] Borkowski TA, Letterio JJ, Farr AG, Udey MC. A role for endogenous transforming growth factor beta 1 in Langerhans cell biology: the skin of transforming growth factor beta 1 null mice is devoid of epidermal Langerhans cells. J Exp Med 1996;184:2417–22. 8976197 10.1084/jem.184.6.2417 PMC2196398

[R7] Borkowski TA, Letterio JJ, Mackall CL, Saitoh A, Wang XJ, Roop DR, A role for TGFbeta1 in langerhans cell biology. Further characterization of the epidermal Langerhans cell defect in TGFbeta1 null mice. J Clin Invest 1997;100:575–81. 9239404 10.1172/JCI119567 PMC508224

[R8] Capucha T, Mizraji G, Segev H, Blecher-Gonen R, Winter D, Khalaileh A, Distinct murine mucosal langerhans cell subsets develop from pre-dendritic cells and monocytes. Immunity 2015;43:369–81. 26231115 10.1016/j.immuni.2015.06.017

[R9] Doi H, Shibata MA, Kiyokane K, Otsuki Y. Downregulation of TGFbeta isoforms and their receptors contributes to keratinocyte hyperproliferation in psoriasis vulgaris. J Dermatol Sci 2003;33:7–16. 14527734 10.1016/s0923-1811(03)00107-5

[R10] Geissmann F, Prost C, Monnet JP, Dy M, Brousse N, Hermine O. Transforming growth factor beta1, in the presence of granulocyte/macrophage colony-stimulating factor and interleukin 4, induces differentiation of human peripheral blood monocytes into dendritic Langerhans cells. J Exp Med 1998;187:961–6. 9500798 10.1084/jem.187.6.961 PMC2212193

[R11] Glitzner E, Korosec A, Brunner PM, Drobits B, Amberg N, Schonthaler HB, Specific roles for dendritic cell subsets during initiation and progression of psoriasis. EMBO Mol Med 2014;6:1312–27. 25216727 10.15252/emmm.201404114 PMC4287934

[R12] Hieronymus T, Zenke M, Baek JH, Seré K. The clash of Langerhans cell homeostasis in skin: should I stay or should I go? Semin Cell Dev Biol 2015;41:30–8. 24613914 10.1016/j.semcdb.2014.02.009

[R13] Hovav AH. Mucosal and skin Langerhans cells - nurture calls. Trends Immunol 2018;39:788–800. 30219310 10.1016/j.it.2018.08.007

[R14] Ito T, Inaba M, Inaba K, Toki J, Sogo S, Iguchi T, A CD1a+/CD11c+ subset of human blood dendritic cells is a direct precursor of Langerhans cells. J Immunol 1999;163:1409–19. 10415041

[R15] Jiang M, Sun Z, Dang E, Li B, Fang H, Li J, TGFβ/SMAD/microRNA-486-3p signaling axis mediates keratin 17 expression and keratinocyte hyperproliferation in psoriasis. J Invest Dermatol 2017;137:2177–86. 28642156 10.1016/j.jid.2017.06.005

[R16] Kaplan DH, Li MO, Jenison MC, Shlomchik WD, Flavell RA, Shlomchik MJ. Autocrine/paracrine TGFbeta1 is required for the development of epidermal Langerhans cells. J Exp Med 2007;204:2545–52. 17938236 10.1084/jem.20071401 PMC2118472

[R17] Kel JM, Girard-Madoux MJ, Reizis B, Clausen BE. TGF-beta is required to maintain the pool of immature Langerhans cells in the epidermis. J Immunol 2010;185:3248–55. 20713882 10.4049/jimmunol.1000981

[R18] Maier B, Leader AM, Chen ST, Tung N, Chang C, LeBerichel J, A conserved dendritic-cell regulatory program limits antitumour immunity [published correction appears in Nature 2020;582:E17]. Nature 2020;580:257–62. 32269339 10.1038/s41586-020-2134-y PMC7787191

[R19] Martínez-Cingolani C, Grandclaudon M, Jeanmougin M, Jouve M, Zollinger R, Soumelis V. Human blood BDCA-1 dendritic cells differentiate into Langerhans-like cells with thymic stromal lymphopoietin and TGF-β. Blood 2014;124:2411–20. 25114264 10.1182/blood-2014-04-568311

[R20] Milne P, Bigley V, Gunawan M, Haniffa M, Collin M. CD1c+ blood dendritic cells have Langerhans cell potential. Blood 2015;125:470–3. 25352125 10.1182/blood-2014-08-593582 PMC4358967

[R21] Mishina Y, Suzuki A, Gilbert DJ, Copeland NG, Jenkins NA, Ueno N, Genomic organization and chromosomal location of the mouse type I BMP-2/4 receptor. Biochem Biophys Res Commun 1995;206:310–7. 7818535 10.1006/bbrc.1995.1043

[R22] Mohammed J, Beura LK, Bobr A, Astry B, Chicoine B, Kashem SW, Stromal cells control the epithelial residence of DCs and memory T cells by regulated activation of TGF-β. Nat Immunol 2016;17:414–21. 26901152 10.1038/ni.3396 PMC5135085

[R23] Nickel J, Mueller TD. Specification of BMP signaling. Cells 2019;8:1579. 31817503 10.3390/cells8121579 PMC6953019

[R24] Régnier M, Patwardhan A, Scheynius A, Schmidt R. Reconstructed human epidermis composed of keratinocytes, melanocytes and Langerhans cells. Med Biol Eng Comput 1998;36:821–4. 10367476 10.1007/BF02518889

[R25] Samavarchi-Tehrani P, Golipour A, David L, Sung HK, Beyer TA, Datti A, Functional genomics reveals a BMP-driven mesenchymal-to-epithelial transition in the initiation of somatic cell reprogramming. Cell Stem Cell 2010;7:64–77. 20621051 10.1016/j.stem.2010.04.015

[R26] Sconocchia T, Hochgerner M, Schwarzenberger E, Tam-Amersdorfer C, Borek I, Benezeder T, Bone morphogenetic protein signaling regulates skin inflammation via modulating dendritic cell function. J Allergy Clin Immunol 2021;147:1810–22.e9. 33250156 10.1016/j.jaci.2020.09.038

[R27] Strobl H, Riedl E, Scheinecker C, Bello-Fernandez C, Pickl WF, Rappersberger K, TGF-beta 1 promotes in vitro development of dendritic cells from CD34+ hemopoietic progenitors. J Immunol 1996;157:1499–507. 8759731

[R28] Tenno M, Shiroguchi K, Muroi S, Kawakami E, Koseki K, Kryukov K, Cbfβ2 deficiency preserves Langerhans cell precursors by lack of selective TGFβ receptor signaling. J Exp Med 2017;214:2933–46. 28814567 10.1084/jem.20170729 PMC5626404

[R29] Wataya-Kaneda M, Hashimoto K, Kato M, Miyazono K, Yoshikawa K. Differential localization of TGF-beta-precursor isotypes in psoriatic human skin. J Dermatol Sci 1996;11:183–8. 8785168 10.1016/0923-1811(95)00438-6

[R30] Yasmin N, Bauer T, Modak M, Wagner K, Schuster C, Köffel R, Identification of bone morphogenetic protein 7 (BMP7) as an instructive factor for human epidermal Langerhans cell differentiation. J Exp Med 2013;210:2597–610. 24190429 10.1084/jem.20130275 PMC3832935

[R31] Yu Q, Parajuli N, Yi Q, Mishina Y, Elder JT, Zhou L, ALK3 is not required for the embryonic development, homeostasis, and repopulation of epidermal Langerhans cells in steady and inflammatory states. J Invest Dermatol 2021;141:1858–61. 33359325 10.1016/j.jid.2020.10.028 PMC8219812

[R32] Yuhki M, Yamada M, Kawano M, Iwasato T, Itohara S, Yoshida H, BMPR1A signaling is necessary for hair follicle cycling and hair shaft differentiation in mice. Development 2004;131:1825–33. 15084466 10.1242/dev.01079

[R33] Zeisberg M, Shah AA, Kalluri R. Bone morphogenic protein-7 induces mesenchymal to epithelial transition in adult renal fibroblasts and facilitates regeneration of injured kidney. J Biol Chem 2005;280:8094–100. 15591043 10.1074/jbc.M413102200

