# Supplementary material for: BMPR1a Is Required for the Optimal TGFβ1-Dependent CD207+ Langerhans Cell Differentiation and Limits Skin Inflammation through CD11c+ Cells
Source: J Invest Dermatol. Author manuscript; Available in PMC 2025 Feb 24. (PMC11849060; doi:10.1016/j.jid.2022.02.014)
Supplement: 1 [file NIHMS2026160-supplement-1.pdf]

SUPPLEMENTARY MATERIALS: SUPPLEMENTARY MATERIALS AND METHODS

Preparation of epidermal single-cell suspensions and flow cytometry analysis

Mice were euthanized, and mouse ears were split into the dorsal and ventral sides and placed on 0.8% trypsin (Gibco, Waltham, MA) for 45 minutes at 37 °C to allow for the separation of epidermis and dermis. The epidermis was cut into small pieces, further digested in 250 μg/ml DNaseI for 30 minutes at 37 °C, washed, and filtered using a 70-μm cell strainer. Single-cell suspensions were subsequently blocked with FC-block (BD Pharmingen, San Diego, CA) and stained with indicated fluorescently labeled antibodies (BioLegend, San Diego, CA) at 4 °C for 30 minutes. Before flow cytometric analysis, SYTOX Blue Dead Cell Stain (Invitrogen, Waltham, MA) was added according to the manufacturer's instructions to identify dead cells. Cells were recorded using an LSR-II flow cytometer (BD Biosciences, Franklin Lakes, NJ) and analyzed using FlowJo software 7.6.4 (Tree Star, Ashland, OR).

Preparation of epidermal sheets

To separate epidermis from dermis, split mouse ears were floated at 37 °C, with the dermal side facing down on 3.5% ammonium thiocyanate for 25 minutes. They were subsequently fixed with 4% paraformaldehyde for 30 minutes at room temperature and further subjected to immunofluorescent staining.

Immunofluorescent microscopy

Epidermal sheets were blocked with 5% goat serum and 2% BSA tris-buffered saline tween-20 (TBS-T) for 1 hour and incubated with primary antibodies diluted in 5% goat serum and 2% BSA TBS-T at 4 °C overnight. Subsequently, epidermal sheets were rinsed and incubated with Hoechst (Sigma-Aldrich, St. Louis, MO) for 30 minutes. Tissue sections were mounted, and pictures were taken using a Nikon Eclipse i80 microscope (Nikon, Tokyo, Japan).

Skin explant cultures

Ears halves from indicated mice were floated on complete medium for the indicated time points at 32 °C, and Langerhans cells were enumerated from epidermal sheets as described. In addition, emigrated cells were harvested from the medium and analyzed by FACS for the indicated marker molecules.

Cell isolation and in vitro differentiation

Human cord blood $CD34^+$ cells were isolated by magnetic sorting using EasySep human CD34-positive selection kit (Stemcell Technologies, Vancouver, British Columbia, Canada) according to the manufacturer's instructions. Cord blood was collected during healthy, full-term deliveries. $CD34^+$ cells were then cultured for 2 days in serum-free X-Vivo media supplemented with Glutmax (Lonza, Basel, Switzerland), penicillin/streptomycin, 50 ng/ml stem cell factor, 50 ng/ml FLT3 ligand, and 50 ng/ml thrombopoietin. For direct Langerhans cell differentiation, $CD34^+$ cells were cultured for 7 days in a 24-well tissue-treated plate (5×10^4 cells per well) in serum-free CellGroDC medium (Cellgenix, Freiburg im Breisgau, Germany) supplemented with Glutmax, penicillin/streptomycin, 2.5 ng/ml TNF-α, 100 ng/ml GM-CSF, 50 ng/ml FLT3 ligand, 20 ng/ml stem cell factor, and either 1 ng/ml TGFβ1 or 200 ng/ml BMP7.

Cytokines and reagents

TNF-α, thrombopoietin, stem cell factor, FLT3 ligand, GM-CSF, and IL-4 were purchased from PeproTech (London, United Kingdom). Human recombinant TGFβ1 was purchased from R&D Systems (Minneapolis, MN). Human recombinant BMP7 was purchased from ImmunoTools (Friesoythe, Germany)

Patient samples

Ethics approval (EK700/2009) was obtained from the Medical University of Vienna (Vienna, Austria) Institutional Review Board for these studies. Written informed consent was provided by patients in accordance with the Declaration of Helsinki. Punch biopsies (4 mm) have been taken from lesional and nonlesional skin of two patients with psoriasis. Skin samples were divided for (i) formalin fixation and immunofluorescence processing of paraffin-embedded material and (ii) tissue digestion for flow cytometry and cell sorting of single-cell suspensions.

Immunofluorescence on human samples

A total of 5 μm paraffin sections were deparaffinized in xylene and rehydrated with decreasing concentrations of ethanol according to the standard method. Sections were subjected to heat-induced epitope retrieval antigen retrieval in Target Retrieval Solution pH 6.0 (Agilent Technologies/Dako, Santa Clara, CA) for 10 minutes in a domestic microwave oven. Slides were allowed to cool for 45 minutes at room temperature before rinsing in TBS-T (pH 7.4). All incubation steps were performed in a dark moist chamber at room temperature. Negative controls were incubated with the appropriate IgG fractions as isotope controls. Sections were blocked with 5% donkey serum 5% BSA in TBS-T for 1 hour before primary antibodies: polyclonal goat anti-human BMPR1A-ALK3 (2.5 μg/ml; R&D Systems) and monoclonal rat anti-human HLA-DR (8.33 μg/ml; Abcam, Cambridge, United Kingdom) were incubated overnight at 4 °C. After 10 minutes of TBS-T wash, secondary antibodies were applied: donkey anti-goat 488 (2.5 μg/ml; clone H+L, Jackson ImmunoReserach Laboratories, West Grove, PA) and donkey anti-rat Cy3 (1.75 μg/ml; clone H+L, Jackson ImmunoReserach Laboratories) for 30 minutes. After rinsing in TBS-T, DAPI was added to the slides for 15 minutes as a nuclei counterstain, then rinsed again with TBS-T before mounting with Dako Fluorescence Mounting Medium (Agilent Technologies, Santa Clara, CA). To acquire and analyze computerized images of sections, a Leica DM4000 B microscope (Leica Microsystems Cambridge, Cambridge, United Kingdom) equipped with a Leica DFC320 Video camera (Leica Microsystems Cambridge) was used.

Flow cytometry of in vitro differentiated cells

Cells were collected, washed, and suspended in 50 μl of PBS and incubated in Fc receptors block for 15 minutes. The staining was performed using the following antibodies: CD1a-BV421 (HI149), CD207-phycoerythrin (DCGM4), BMPR1a-allophycocyanin (MAB2406). Flow cytometry data were collected by using the LSRFortessa (BD Biosciences).

Analysis was conducted using the DIVA (BD Biosciences) and FlowJo software.

Statistics

Statistical comparisons were performed using GraphPad Prism 5.02 software (GraphPad Software, San Diego, CA). Statistical significance was determined using Student's unpaired two-tailed *t*-test for comparisons of two groups and one-way ANOVA followed by Tukey's posthoc test for multiple comparisons. Experiments were repeated independently at least two times with similar results. Dot plots depict the biological replicates unless otherwise stated. Data are represented as means ± SEM; n describes the number of biological replicates.

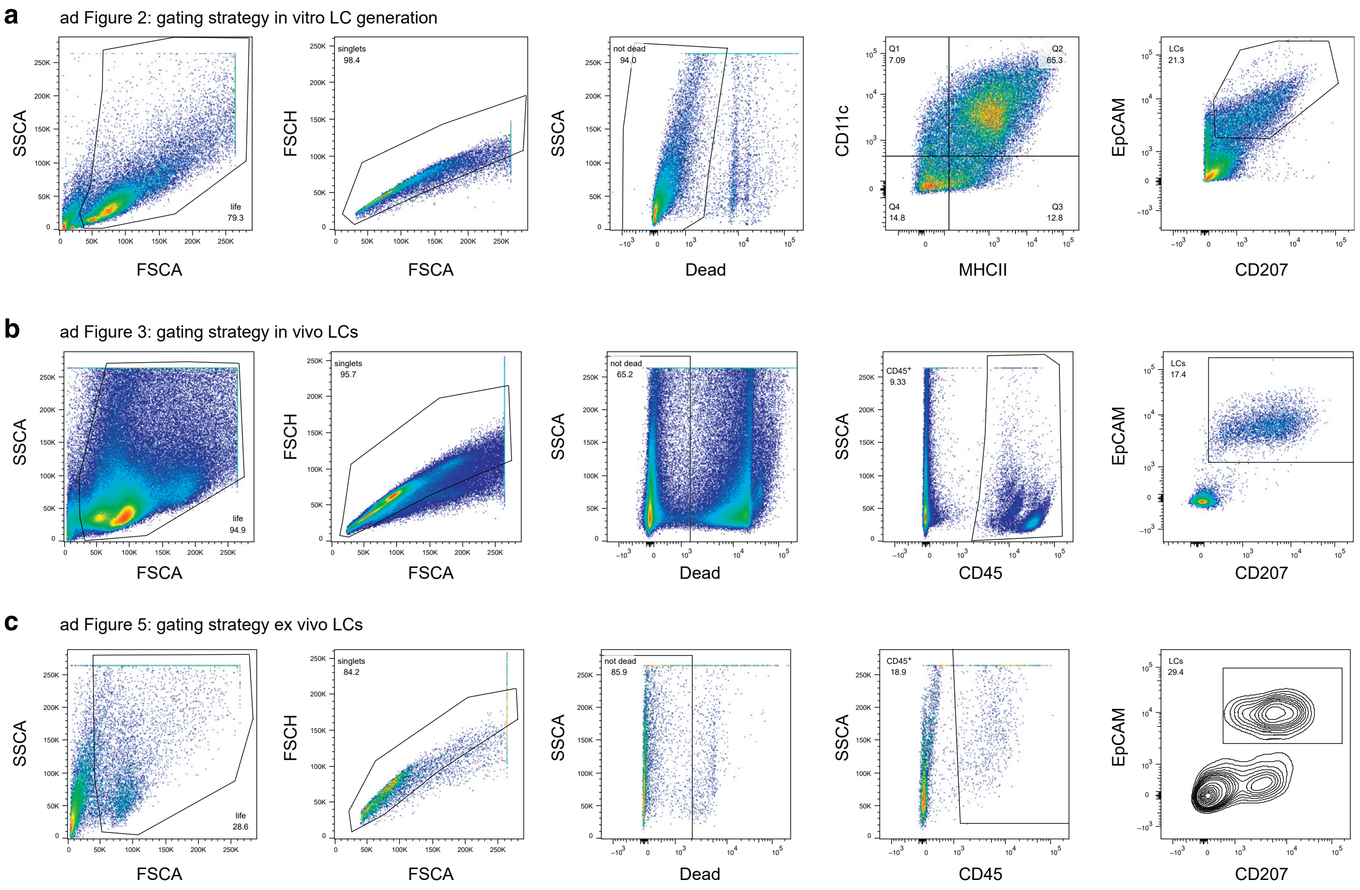

Supplementary Figure S1. Gating strategy of indicated FACS analysis data. (**a**) Gating strategy for in vitro generated LCs from bone marrow cultures as used in Figure 2. (**b**) Gating strategy for in vivo LCs from mouse epidermal cell suspensions as used in Figure 3. (**c**) Gating strategy for ex vivo emigrated LCs from skin explant cultures as used in Figure 5. ad, to; FSCA, forward scatter area; FSCH, forward scatter height; K, thousand; LC, Langerhans cell; Q, quarter; SSCA, side scatter area.

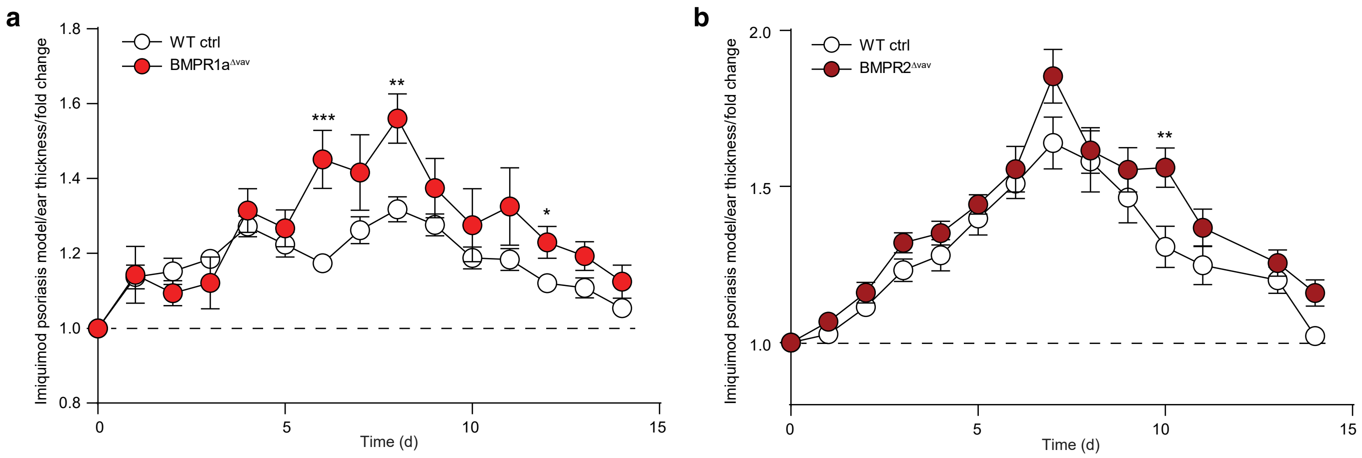

Supplementary Figure S2. Assessment of imiquimod-induced skin inflammation in BMPR1a$^{\Delta vav}$ and BMPR2$^{\Delta vav}$ mice. (**a**) Imiquimod skin inflammation assay was performed on WT and BMPR2$^{\Delta vav}$ mice. Ear swelling was monitored for the indicated time points; shown as mean ± SEM. $^{**}P < 0.01$ is determined by Student's *t*-test for each time point. (**b**) Imiquimod skin inflammation assay was performed on WT and BMPR1a$^{\Delta vav}$ mice. Ear swelling was monitored for the indicated time points; shown as mean ± SEM. n ≥ 4 per group and time point. The graph represents data from at least two independent experiments. $^{*}P < 0.05$, $^{**}P < 0.01$, and $^{***}P < 0.001$ are determined by Student's *t*-test for each time point. ctrl, control; d, day; WT, wild type.
